# Demographic and Clinical Correlates of Body Mass Index in Older Age Bipolar Disorder: Results from the GAGE-BD Project

**DOI:** 10.3390/medicina62040761

**Published:** 2026-04-15

**Authors:** Carol K. Chan, Kasia G. Rothenberg, Farren B. S. Briggs, Amulya Mallu, Nicole M. Fiorelli, Osvaldo P. Almeida, Kürşat Altınbaş, Vicent Balanzá-Martinez, Izabela G. Barbosa, Hilary P. Blumberg, Peijun Chen, Leon Flicker, Ariel Gildengers, Bartholomeus C. M. Haarman, Federica Klaus, Beny Lafer, Paula V. Nunes, Andrew T. Olagunju, Stephen O. Oluwaniyi, Melis Orhan, Regan E. Patrick, Edith Pomarol-Clotet, Joaquim Radua, Soham Rej, Jair C. Saores, Antonio L. Teixeira, Shangying Tsai, Sonia L. L. Vidal-Rubio, Eduard Vieta, Sara L. Weisenbach, Lisa T. Eyler, Annemiek Dols, Martha Sajatovic

**Affiliations:** 1 Center for Brain Health, Neurological Institute, Cleveland Clinic, Cleveland, OH 44195, USA; 2 Department of Public Health Sciences, Miller School of Medicine, University of Miami, Miami, FL 33136, USA; 3 School of Medicine, Case Western Reserve University, Cleveland, OH 44106, USA; 4 Institute for Health Research, University of Notre Dame Australia, Fremantle 6160, Australia; 5 Medical School, University of Western Australia, Crawley 6009, Australia; 6 Department of Psychiatry, Atlas University, 34212 Istanbul, Turkey; 7 Teaching Unit of Psychiatry and Psychological Medicine, Department of Medicine, University of Valencia, CIBERSAM, 46010 Valencia, Spain; 8 Departamento de Psiquiatria, Faculdade de Medicina, Universidade Federal de Minas Gerais, Belo Horizonte 31270-901, MG, Brazil; 9 Department of Psychiatry, Yale School of Medicine, 60, New Haven, CT 06520, USA; 10 Department of Psychiatry, University Hospitals Cleveland Medical Center, Cleveland, OH 44106, USA; 11 Geriatric Research, Education and Clinical Center (GRECC), VA Northeast Ohio Healthcare System, Cleveland, OH 44106, USA; 12 Western Australian Center for Health and Ageing, Medical School, University of Western Australia, Perth 6009, Australia; 13 Department of Psychiatry, University of Pittsburgh, Pittsburgh, PA 15213, USA; 14 Department of Psychiatry, University Medical Center Groningen, University of Groningen, 9700 RB Groningen, The Netherlands; 15 Department of Psychiatry, University of California, San Diego, CA 92103, USA; 16 Department of Psychiatry, University of São Paulo Medical School, São Paulo 05403-903, SP, Brazil; 17 Department of Psychiatry and Behavioral Neurosciences, McMaster University, Hamilton, ON L8N 3K7, Canada; 18 St. Joseph’s Healthcare Hamilton, Hamilton, ON L8N 3K7, Canada; 19 Discipline of Psychiatry, University of Adelaide, Adelaide 5000, Australia; 20 Federal Neuro-Psychiatric Hospital Yaba, Lagos 101245, Nigeria; 21 GGZ inGeest, Specialized Mental Health Care, 1070 BB Amsterdam, The Netherlands; 22 Departments of Neuropsychology & Geriatric Psychiatry, McLean Hospital, Belmont, MA 02478, USA; 23 Department of Psychiatry, Harvard Medical School, Belmont, MA 02478, USA; 24 Fundació per a la Investigació i Docència Maria Angustias Giménez (FIDMAG) Germanes Hospitalàries Research Foundation, 08830 Barcelona, Spain; 25 Centro de Investigación Biomédica en Red de Salud Mental (CIBERSAM), Instituto de Salud Carlos III (ISCIII), 28029 Madrid, Spain; 26 Institut d’Investigacions Biomèdiques August Pi i Sunyer (IDIBAPS), Centro de Investigación Biomédica en Red de Salud Mental (CIBERSAM), Institute of Neurospascience, University of Barcelona, 08035 Barcelona, Spain; 27 School of Nursing and Midwifery, Queen’s University Belfast, Belfast BT9 7BL, UK; 28 Department of Psychiatry and Behavioral Sciences, University of Texas Health Science Center at Houston, Houston, TX 77030, USA; 29 Department of Psychiatry, Taipei Medical University and Taipei Medical University Hospital, Taipei 110301, Taiwan; 30 Psychiatry Service, Hospital de la Ribera, 46600 Alzira, Spain; 31 Hospital Clinic, Institute of Neuroscience, University of Barcelona, 08036 Barcelona, Spain; 32 Department of Psychiatry, University Medical Center Utrecht, 3584 CX Utrecht, The Netherlands; 33 University Hospitals of Cleveland, Cleveland, OH 44106, USA

**Keywords:** bipolar disorder, older age, obesity, overweight, BMI, geriatric

## Abstract

*Background and Objectives*: There are known associations between bipolar disorder and obesity, but it has not been well characterized in older adults with bipolar disorder (OABD). This study aims to examine body mass index (BMI) and its clinical correlations in OABD. *Materials and Methods*: A secondary analysis was conducted using data from the Global Aging and Geriatric Experiments in Bipolar Disorder (GAGE-BD) project, an international harmonized dataset of OABD cohorts. To examine the relationship between BMI and clinical characteristics (e.g., sex, psychiatric history, symptom severity, medication use, comorbidities), multivariable linear regression and multinomial logistic regression models with random effect for study cohort were used, with BMI as a continuous and as an ordinal (underweight vs. healthy weight vs. overweight vs. obese) dependent variable, respectively. *Results*: Of 1,226 OABD participants with BMI data, 405 (33.0%) were classified as overweight (BMI 25–29.99) and 462 (37.7%) as obese (BMI > 30). In linear regression models, higher BMI was associated with younger age, higher number of somatic comorbidities, and anticonvulsant use, while lower BMI was associated with lithium use. In logistic regression models, obesity was associated with cardiovascular comorbidity, musculoskeletal comorbidity and endocrine comorbidity. *Conclusions*: A high proportion of individuals with OABD are overweight or obese. Several demographic and clinical correlations of higher BMI were found, including younger age, higher number of medical comorbidities and anticonvulsant use. Clinicians should monitor and manage weight changes and associated comorbidities, and promote lifestyle and health interventions to minimize the risk of negative health outcomes associated with high BMI.

## 1. Introduction

Globally, the prevalence of obesity has increased since the 1990s, and the number of older adults with bipolar disorder (BD) is also projected to increase [1]. The association between several mental disorders and high body mass index (BMI) is well described, although the direction of this relationship remains unclear [2,3,4], with some studies suggesting a reciprocal relationship between obesity and BD [5,6,7]. Older adults with BD also have been noted to have high BMIs compared to healthy controls [8]. Despite this link, not much is known about factors relating to higher BMI in BD, and BMI in general is not well-studied in older age BD (OABD), defined as BD in individuals greater than 50 years old [4]. These limitations were highlighted in a 2023 systematic review on physical health comorbidities in OABD, where it was noted that there were no recent studies on predictors of obesity in OABD [1].

The association between BMI, age, and clinical characteristics remains unclear. Some have noted that older age is associated with higher BMI [8,9] while others have found no association [10]. In addition, higher BMI has been associated with longer duration of illness [8,10]. In some studies, female sex has been associated with higher BMI [9,11,12,13] while others observed that male sex was associated with higher BMI [8]. Psychotropic medications have also been associated with higher BMI. Some have attributed the risk specifically to the use/prescription of second-generation antipsychotics and mood stabilizers among individuals with BD [12,14] while others have observed that even drug naïve individuals with BD are at greater risk for overweight and obesity [15]. To our knowledge, however, these correlations have not been specifically studied in OABD.

Obesity is, in itself, a well-established risk factor for numerous health conditions [16]. When co-occurring with BD, elevated BMI has been linked to increased depressive symptoms [9,17,18], albeit not consistently. Stenzel et al., in their cross-sectional study, observed an inverse relationship between BMI and suicidality [19] prompting the need for deeper inquiry into the nature of this association. Regarding manic episodes, some investigations report a positive correlation between higher BMI and increased manic episodes [17], while others failed to replicate this association [9].

Beyond psychiatric outcomes, higher BMI in BD is also associated with somatic comorbidities such as diabetes [9,10,11], hyperlipidemia [19], hypertension [10], arthritis [11], and worse cognitive functioning [10,20,21]. Furthermore, obesity in BD has been linked to worse social outcomes such as overall functioning [22], lower education level [18] and unemployment [10].

Given the very limited literature on BMI correlates in OABD, the Global Aging and Geriatric Experiments in Bipolar Disorder Database (GAGE-BD), the largest integrated dataset specific to OABD, provides an opportunity to assess correlates of BMI and aging in BD. The aims of our analyses, derived from the GAGE-BD, were to (1) examine the association between BMI and demographic characteristics, including age, sex, education, employment, and (2) investigate whether higher BMI in OABD was associated with clinical variables, such as age of onset, psychiatric hospitalizations, mania and depression severity, psychiatric and somatic comorbidity burden, and functioning.

## 2. Methods

### 2.1. GAGE-BD Study Design

Data for these analyses were derived from the GAGE-BD collaboration, a harmonized dataset of cross-sectional information for outpatients with OABD across multiple international study sites. Methodological details have been previously described [23]. Briefly, the GAGE-BD integrated database contains de-identified data from archival research and clinical studies of OABD samples. Approval to contribute data was obtained from each site’s institutional review board or ethics committee. Demographic characteristics (age, sex, education, and employment status) and clinical data (diagnosis, BD subtype, age of onset, depression severity, and mania severity) were harmonized across studies [24,25,26]. Details on sample characteristics and metadata about the contributing studies are described in Supplemental Table S1.

The analysis used baseline, cross-sectional data from Wave 1 and 2 of the GAGE-BD integrated dataset. It included participants >50 years old with BMI data. A total of 1226 participants from 30 studies spanning 18 sites contributed data to this analysis (2 sites from East Asia and the Pacific, 5 from Europe and Central Asia, 2 from Latin America and the Caribbean, 8 from North America, and 1 from Sub-Saharan Africa).

### 2.2. Outcome Measures

BMI was analyzed as both a continuous and ordinal variable (underweight < 18.5, healthy weight 18.5–24.9, overweight 25–29.9, obese > 30 [kg/m^2^]). Data on BD illness severity, including the number of lifetime psychiatric hospitalizations, excluding admissions for substance use or dependence, were examined whenever available. Information on comorbid substance use disorders and anxiety disorders were categorized as lifetime or current. Demographic measures included age, self-reported sex, and education (in years completed).

Available baseline psychiatric symptom measures included the Young Mania Rating Scale (YMRS) [27], an 11-item clinician-rated scale with a maximum score of 60, used to assess the severity of manic symptoms over the prior 48 h. A higher total score corresponds to a higher severity of symptoms. Depressive symptom severity was categorized into three ordinal severity bands (absent, mild to moderate, severe) based on scores on the Hamilton Depression Rating Scale (HAM-D) [28], Montgomery–Asberg Depression Rating Scale (MADRS) [29], and the Center for Epidemiological Studies—Depression (CES-D) [30]. The process for categorizing depressive symptoms from the GAGE-BD collaboration into depression symptom bands have previously been described in detail [24,25].

Functioning was measured by employment status (currently employed vs. not) and the Global Assessment of Functioning (GAF) score [31]. The GAF is a clinician-rated scale between 0 and 100. It measures the impact of an individual’s psychiatric symptoms on their day-to-day functioning, where a higher score corresponds to a lower impact of psychiatric symptoms on functioning.

Somatic comorbidities were available in the GAGE-BD dataset. They had been harmonized from standardized evaluations such as the Charlson Comorbidity Index [32], the Cumulative Illness Rating Scale for Geriatrics [33], and/or medical comorbidity categories derived from the assessment of study participants. Somatic data was available as dichotomous variables (somatic comorbidity present vs. absent) in the following systems: cardiovascular, respiratory, gastrointestinal, hepatic/pancreatic, renal, genitourinary, musculoskeletal, and endocrine.

Current psychotropic medication use was extracted and categorized by class, including antipsychotic, long-acting injectable antipsychotic, lithium, anticonvulsant, and antidepressant. Antipsychotic medication had been further categorized as first generation, second generation, or both.

### 2.3. Statistical Analysis

Descriptive statistics across BMI groups were compared using Fisher’s exact test for categorical variables and Kruskal–Wallis test for ordinal numerical measures. To examine the relationship between BMI and clinical characteristics, multivariable linear regression and multinomial logistic regression models with random effect for study cohort were used, with BMI as a continuous and as an ordinal dependent variable, respectively.

For multivariable linear regression models examining BMI as a continuous variable, the baseline model included age, sex, and education. Due to the large number of variables of interest, and because complete data was not available across all observations, we iteratively examined the relationships for the following variables adjusting for baseline predictors: symptom severity, functional status, somatic comorbidities, and psychotropic medication use. Model 2 included variables related to symptom severity, including YMRS and depression band scores. Model 3 added variables related to functioning, including GAF score and employment status to the baseline model. Model 4 added the number of somatic comorbidities to the baseline model. Finally, Model 5 added variables related to psychotropic medication use, including the use of first-generation antipsychotics, second-generation antipsychotics, more than one antipsychotic, lithium, anticonvulsants, and antidepressants to the baseline model.

Multinomial logistic regression models were used to examine BMI as a categorical variable. Underweight and healthy weight were combined and used as the reference category because there were only 12 underweight participants. Only variables with significant differences across BMI groups from descriptive comparisons were included in this model. BPRS was excluded from the model as it was only available in 81 subjects. Although renal comorbidity was significantly different across BMI groups, it was excluded from the multinomial logistic regression model due to low frequency (6.72%).

To explore differences across geographical regions, the percentage of OABD falling into each BMI group was calculated separately across East Asia and Pacific, Europe and Central Asia, Latin America and the Caribbean, North America, and Sub-Saharan Africa cohorts.

Missing data was handled using complete case analysis. Statistical significance was defined as a two-sided *p* < 0.05. All analyses were completed using STATA v17 (StataCorp, College Station, TX, USA).

## 3. Results

A total of 1226 participants over 50 years old were included in these analyses (Table 1), with a mean age of 62.57 (SD = 8.07). In total, 359 (29.3%) of them had a BMI of less than 24.99 and were categorized as underweight and healthy weight. As only 12 individuals were categorized as underweight, the underweight and healthy weight participants were grouped together. A BMI between 25.00 and 29.99 was categorized as overweight, representing 405 (33.0%) participants. A BMI of 30 or higher was categorized as obese, present in 462 (37.7%) participants. In multivariable linear regression models, significant differences across BMI groups were observed for age, sex, age of onset, years of education, employment status, depressive symptom severity, BRPS (24 items), cardiovascular comorbidity, respiratory comorbidity, renal comorbidity, musculoskeletal comorbidity, endocrine comorbidity, antipsychotic use, lithium use, and anticonvulsant use.

Across the five multivariable linear regression models (Table 2), older age was consistently associated with lower BMI. In Model 4, a higher number of somatic comorbidities were associated with higher BMI. In Model 5, anticonvulsant use was associated with higher BMI while lithium use was associated with lower BMI. Of note, across all models there was no evidence for differences in BMI by sex or education level. There were also no significant associations with BMI when examining YMRS total score, depressive severity, measures of function (GAF, employment status), antipsychotic use, or antidepressant use.

In the multinomial logistic regression model (Table 3), OABD with endocrine comorbidities was associated with higher odds of being in the overweight (OR = 1.79, *p* = 0.006) and obese (OR = 2.13, *p* < 0.001) categories. Those with higher YMRS were more likely to be healthy or underweight than overweight (OR = 0.98, *p* = 0.04). OABD with cardiovascular and/or musculoskeletal comorbidities were more likely to be obese compared to healthy/underweight (cardiovascular: OR = 2.26, *p* < 0.001; musculoskeletal: OR = 1.63, *p* = 0.02). No significant independent associations were observed for sex, education, age of onset, employment status, depressive symptom burden, respiratory comorbidity burden or lithium use in these models. Results were unchanged when the 12 underweight participants were excluded.

**Table 3:** Multinomial logistic regression with random effect for study cohort for BMI. N = 882. Reference = underweight/healthy weight.

Predictor	Overweight: BMI 25.00–29.99		Obese: BMI 30.00 or Higher	
	OR (95% CI)	*p*	OR (95% CI)	*p*
Age	1.00 (0.97, 1.03)	0.79	0.97 (0.94, 1.00)	0.058
Female	0.76 (0.53, 1.09)	0.13	0.94 (0.65, 1.35)	0.74
College educated	1.08 (0.72, 1.61)	0.71	0.81 (0.53, 1.25)	0.35
Age of onset	1.00 (0.98, 1.01)	0.60	0.99 (0.98, 1.01)	0.27
Employed	1.07 (0.66, 1.73)	0.79	0.82 (0.50, 1.33)	0.42
Depression moderate vs. mild	1.25 (0.83, 1.89)	0.29	1.26 (0.82, 1.95)	0.29
Depression high vs. mild	0.79 (0.34, 1.84)	0.58	0.92 (0.35, 2.41)	0.87
YMRS total score	0.98 (0.96, 1.00)	0.039 *	1.00 (0.98, 1.02)	0.94
Cardiovascular comorbidity	1.35 (0.90, 2.02)	0.15	2.26 (1.49, 3.42)	<0.001 *
Respiratory comorbidity	0.73 (0.48, 1.13)	0.16	1.38 (0.90, 2.13)	0.14
Musculoskeletal comorbidity	1.21 (0.81, 1.82)	0.35	1.63 (1.08, 2.47)	0.02 *
Endocrine comorbidity	1.79 (1.18, 2.70)	0.006 *	2.13 (1.41, 3.21)	<0.001 *
Lithium	0.68 (0.43, 1.08)	0.10	1.13 (0.71, 1.80)	0.62
Anticonvulsant	1.28 (0.84, 1.95)	0.25	1.47 (0.97, 2.22)	0.071

Only variables with *p* < 0.05 in Table 1 were included. BPRS total 24 was not considered because it was only available in 81 subjects. Indicator variables were added to capture missing values for categorical variables. The symbol * signifies significance *p* < 0.05. OR = odds ratio, CI = confidence interval.

Across geographical regions, North American participants had the highest mean BMI at 30.16 (SD = 6.73), while participants from Sub-Saharan Africa had the lowest mean BMI at 24.10 (SD = 3.60) (Table 4).

**Table 4:** BMI in GAGE-BD sample > 50 by geographical region.

Geographical Region	All Cases (N)	Average BMI (M|SD)	Underweight and Healthy Weight: BMI Less Than 24.99	Overweight: BMI 25.00–29.99	Obese: BMI 30.00 or Higher
East Asia and Pacific	156	26.74 (4.25)	59 (37.82%)	62 (39.74%)	35 (22.44%)
Europe and Central Asia	237	28.00 (5.83)	78 (32.91%)	89 (37.55%)	70 (29.54%)
Latin America and the Caribbean	69	27.94 (5.61)	22 (31.88%)	21 (30.43%)	26 (37.68%)
North America	720	30.16 (6.73)	168 (23.33%)	226 (31.39%)	326 (45.28%)
Sub-Saharan Africa	44	24.10 (3.60)	32 (72.73%)	7 (15.91%)	5 (11.36%)

N = number; M = mean; SD = standard deviation.

## 4. Discussion

In this cross-sectional analysis of an international cohort of outpatients diagnosed with OABD, over one-third of the sample met criteria for obesity. This finding underscores the critical importance of integrating routine weight surveillance and structured interventions into standard psychiatric care for this population. Our finding aligns with a growing body of literature indicating that a substantial proportion of OABD are affected by obesity, highlighting the need to prioritize weight management not merely as a lifestyle issue but as a fundamental clinical concern [1,8].

With respect to demographic and clinical correlates, we observed that higher BMI in OABD was associated with younger age, somatic comorbidity, and anticonvulsant use when examined as a continuous variable. Lithium use was associated with lower BMI when BMI was examined as a continuous variable, but no significant association was observed when analyzed as a categorical variable. No significant association was observed with antidepressant use or antipsychotic use.

Similar to patterns in the general population, we observed that younger age was associated with higher BMI [34]. Overall, the proportion of participants in the GAGE-BD cohort with BMI ≥ 30 (37.7%) was largely consistent with rates of obesity in other studies of individuals with BD [8,17]. Unlike prior studies of younger cohorts with BD, we did not observe an association between higher BMI and depressive symptom severity [9,17,35]. Studies in older adults in the general population in Mexico, Korea and Taiwan have reported an association between lower BMI and more depressive symptoms, particularly for women [36,37,38]. A large US-based study reported the contrary, observing that higher BMI was associated with depression in the older population [39]. Such differences may be, in part, attributable to cultural differences relating to lifestyle behaviors and attitudes around weight and may have contributed to our observations in an international cohort [40].

Mood stabilizers, including anticonvulsants, lithium, and antipsychotic agents, remain central to the management of BD. Among these, second-generation antipsychotics (SGAs) are widely used but are consistently associated with metabolic adverse effects, including weight gain and higher BMI, in both BD [12,14,41] and schizophrenia [42]. In older adults, the relationship between SGAs and weight gain seems less pronounced. Several studies have shown that SGA-related weight changes in older patients are typically smaller than those seen in younger populations [43,44,45], suggesting age-related variations in metabolic response.

While we did not observe an association between antipsychotic use and BMI in our OABD cohort, we observed that anticonvulsants were associated with higher BMI. This finding must be interpreted in light of the considerable heterogeneity among anticonvulsants, as individual agents may exert differential effects on weight [46]. For example, valproic acid has been associated with higher risk of weight gain, as opposed to other anticonvulsants such as lamotrigine or topiramate that are typically considered weight-neutral or may even promote weight loss [47]. Furthermore, due to the cross-sectional nature of our analyses, we were not able to account for potential biases in prescribing patterns. For example, patients who were already overweight may be less likely to be prescribed an atypical antipsychotic due to concerns of further weight gain. Regional prescribing practices and socioeconomic factors may also affect patterns of medication use. Our observation that lithium was associated with lower weight is largely consistent with the existing literature. A 2022 meta-analysis of nine studies did not observe any significant changes in weight in patients treated with lithium [48] and found that most studies included did not observe greater weight gain in lithium-treated groups compared to placebo.

Elevated BMI in BD has been consistently associated with a broad array of somatic comorbidities, including but not limited to type 2 diabetes [9,10,11], hyperlipidemia [19], hypertension [10], and arthritis [11]. These associations reflect not only an increased burden of physical illness in this population but also point to the bidirectional nature of psychiatric and metabolic dysfunction in BD.

Moreover, higher BMI has been linked to worse neurocognitive outcomes in BD, with several studies reporting significant associations between obesity and reduced cognitive performance across domains such as executive functioning, memory, and processing speed [10,20,21]. Older patients with BD have greater risk of cognitive impairment, and greater risk has been associated with lower education, type I BD, and somatic comorbidities [49]. In addition, social and functional correlates of obesity have been documented, including associations with lower psychosocial functioning [50] and educational attainment [18], which may reflect cumulative disadvantages across the lifespan. Although some investigations suggest that obesity is more prevalent among individuals with BD who are unemployed or are receiving disability benefits [10], others have failed to replicate this association [51], indicating the complexity of underlying factors and the necessity for more research.

Given these multifaceted somatic, cognitive, and psychosocial risks, it is imperative that patients with OABD be systematically counseled on the adverse health implications of obesity. Treatment planning should incorporate individualized weight management strategies that are both medically sound and tailored to each patient’s cognitive and functional capacities. Previous analyses emphasize individuals with OABD, particularly those with high physical or somatic burden, may be at heightened risk of pharmacological side effects and should therefore receive particularly close clinical surveillance [52]. Our data support and extend these recommendations, confirming that younger individuals within the OABD cohort—especially those with significant comorbid physical conditions—exhibit elevated vulnerability, thereby reinforcing the need for proactive interventions. This includes the preferential use of psychotropic agents with lower metabolic liability and the implementation of integrated care models that promote coordinated management across psychiatric, primary care, and specialty medical services [52].

Our findings should be interpreted within the context of methodological limitations. The study was cross-sectional, not allowing to infer causality. The inclusion of only variables with significant descriptive statistics in the multinomial logistic regression models was done so to reduce the risk of overfitting, given the large number of variables. How-ever, we acknowledge that this methodology could risk model misspecification. We did not examine factors such as family history, diet, physical activity, and exposure to other medications that could affect BMI. We only included major drug classes, which may have heterogenous mechanisms of action and side effect profiles. Furthermore, we did not have data on comorbid eating disorders, which has been observed in 14.3% of BD patients [53], and may also contribute to BMI differences in some patients. There are inherent challenges associated with the use of harmonized data collected at different times and contexts. That said, the GAGE-BD cohort provides a large dataset of OABD, expanding on prior studies on BMI and BD.

## 5. Conclusions

In sum, in an international cohort of outpatients with OABD, higher BMI was associated with younger age, somatic comorbidity, and anticonvulsant use. Future studies should consider these factors when evaluating the relationship between OABD and weight. Given the high prevalence of obesity and multimorbidity in OABD, treatment strategies for this population should be specifically studied rather than inferred from data on younger adults. Further research is needed to clarify how elevated BMI and physical health comorbidities influence pharmacologic efficacy, tolerability and long-term outcomes in OABD.

## Figures and Tables

**Table 1:** Descriptive statistics for GAGE-BD sample > 50 years old by BMI groups.

Variables	All Cases (N = 1226)	Underweight and Healthy Weight: BMI Less Than 24.99 (n = 359)	Overweight: BMI 25.00–29.99 (n = 405)	Obese: BMI 30.00 or Higher (n = 462)	*p*-Value
BMI	28.97 (6.34)	22.43 (1.81)	27.35 (1.40)	35.46 (4.96)	
BMI Range	15.59–67.13	15.59–24.98	25.00–29.98	30.00–67.13	
Age in Years	62.57 (8.07)	63.21 (8.84)	63.27 (8.07)	61.45 (7.30)	0.003 *
Age Range in Years	51.00–90.00	51.00–87.40	51.00–90.00	51.00–85.00	
Female	657 (53.59%)	196 (54.60%)	195 (48.15%)	266 (57.58%)	0.019 *
Diagnosis					
Bipolar I	956 (83.35%)	280 (29.29%)	304 (31.80%)	372 (38.91%)	0.491
Bipolar II	184 (16.04%)	52 (28.26%)	65 (35.33%)	67 (36.41%)	
Unknown/NOS	7 (0.61%)	1 (14.29%)	1 (14.29%)	5 (71.43%)	
Age of Onset in Years	32.64 (15.68)	34.15 (16.75)	33.32 (16.10)	30.74 (14.11)	0.048 *
Years of Education	12.90 (4.25)	12.63 (4.38)	13.45 (4.34)	12.64 (4.02)	0.045 *
GAF Total	55.46 (18.33)	54.20 (21.50)	57.95 (17.19)	54.27 (15.90)	0.062
Employed	217 (25.44%)	73 (30.54%)	72 (26.47%)	72 (21.05%)	0.031 *
Psychiatric Hospitalizations					
Yes	417 (69.04%)	126 (68.11%)	139 (70.56%)	152 (68.47%)	0.857
No	187 (30.96%)	59 (31.89%)	58 (29.44%)	70 (31.53%)	
Number of Psychiatric Hospitalizations	2.72 (4.31)	2.02 (2.61)	3.11 (5.33)	2.97 (4.41)	0.307
Depression Band					
Absent	527 (48.80%)	166 (54.07%)	174 (49.71%)	187 (44.21%)	0.012 *
Mild to Moderate	496 (45.93%)	120 (39.09%)	157 (44.86%)	219 (51.77%)	
Severe	57 (5.28%)	21 (6.84%)	19 (5.43%)	17 (4.02%)	
YMRS Total	9.35 (11.65)	9.91 (12.98)	8.56 (10.91)	9.59 (11.12)	0.119
BPRS Total (18 Items)	29.43 (14.55)	28.12 (16.75)	27.88 (14.37)	31.23 (12.78)	0.352
BPRS Total (24 Items)	45.33 (12.18)	49.57 (12.75)	39.58 (9.82)	44.40 (11.09)	0.010 *
Somatic Comorbidity					
Cardiovascular	576 (50.22%)	138 (41.95%)	185 (48.81%)	253 (57.63%)	<0.001 *
Respiratory	438 (39.57%)	116 (36.02%)	129 (35.34%)	193 (45.95%)	0.003 *
Gastrointestinal	279 (27.51%)	71 (24.74%)	95 (28.79%)	113 (28.46%)	0.465
Hepatic or Pancreatic	78 (8.49%)	24 (8.89%)	24 (7.97%)	30 (8.62%)	0.922
Renal	60 (6.72%)	10 (4.22%)	29 (10.25%)	21 (5.63%)	0.016 *
Genitourinary	198 (26.36%)	60 (28.44%)	65 (26.10%)	73 (25.09%)	0.693
Musculoskeletal	468 (46.71%)	103 (36.27%)	143 (43.87%)	222 (56.63%)	<0.001 *
Endocrine	422 (37.85%)	82 (25.23%)	135 (36.00%)	205 (49.40%)	<0.001 *
Current Non-Bipolar Psychiatric Diagnosis					
None	494 (67.67%)	148 (69.16%)	154 (65.81%)	192 (68.09%)	0.509
Anxiety Disorder	148 (20.27%)	39 (18.22%)	46 (19.66%)	63 (22.34%)	
Substance or Alcohol Use Disorder	65 (8.90%)	20 (9.35%)	27 (11.54%)	18 (6.38%)	
Both	23 (3.15%)	7 (3.27%)	7 (2.99%)	9 (3.19%)	
Lifetime Non-Bipolar Comorbid Psychiatric Diagnosis					
None	330 (38.64%)	109 (43.78%)	94 (34.56%)	127 (38.14%)	0.43
Anxiety Disorder	217 (25.41%)	55 (22.09%)	69 (25.37%)	93 (27.93%)	
Substance or Alcohol Use Disorder	177 (20.73%)	49 (19.68%)	64 (23.53%)	64 (19.22%)	
Both	130 (15.22%)	36 (14.46%)	45 (16.54%)	49 (14.71%)	
Current Psychotropic Medication Use (Antipsychotic Generations)					
First Generation	40 (9.05%)	20 (14.81%)	14 (10.53%)	6 (3.45%)	0.008 *
Second Generation	384 (86.88%)	110 (81.48%)	113 (84.96%)	161 (92.53%)	
Both Generations	18 (4.08%)	5 (3.70%)	6 (4.51%)	7 (4.02%)	
Current Psychotropic Medication Use					
Antipsychotic	493 (45.44%)	146 (47.10%)	148 (43.27%)	199 (45.96%)	0.592
>1 Antipsychotic	33 (10.44%)	7 (7.95%)	10 (10.20%)	16 (12.31%)	0.603
Long-Acting Injectable Antipsychotic	2 (3.28%)	2 (9.09%)	0 (0.00%)	0 (0.00%)	0.327
Lithium	299 (28.61%)	97 (34.52%)	95 (28.36%)	107 (24.94%)	0.023 *
Anticonvulsant	408 (39.12%)	90 (32.14%)	139 (41.49%)	179 (41.82%)	0.019 *
Antidepressant	344 (36.83%)	81 (32.40%)	108 (35.41%)	155 (40.90%)	0.081

Note: Current non-bipolar psychiatric diagnosis and lifetime non-bipolar psychiatric diagnosis examined only the participants who have data on both anxiety disorders and substance- or alcohol-use disorders. To determine the *p* values, the Kruskal–Wallis test was used for continuous variables, and Fisher’s exact test was used for categorical variables. The symbol * signifies significance *p* < 0.05.

**Table 2:** Multivariable linear regression for BMI with study cohort as a random effect.

Predictor	Model 1		Model 2		Model 3		Model 4		Model 5	
	β (95% CI)	*p*	β (95% CI)	*p*	β (95% CI)	*p*	β (95% CI)	*p*	β (95% CI)	*p*
N	1226		1032		672		679		357	
Age	−0.015 (−0.023, −0.007)	<0.001 *	−0.013 (−0.022, −0.004)	<0.001 *	−0.017 (−0.028, −0.006)	<0.001 *	−0.021 (−0.031, −0.011)	<0.001 *	−0.022 (−0.037, −0.007)	0.004 *
Female	0.024 (−0.087, 0.135)	0.68	−0.028 (−0.149, 0.094)	0.66	0.087 (−0.066, 0.24)	0.27	0.000 (−0.145, 0.146)	0.99	0.066 (−0.155, 0.286)	0.56
College educated	−0.128 (−0.270, 0.013)	0.075	−0.124 (−0.273, 0.026)	0.11	−0.096 (−0.271, 0.079)	0.28	−0.121 (−0.290, 0.049)	0.16	−0.185 (−0.448, 0.079)	0.17
Current Symptoms										
YMRS Total Score			0.000 (−0.007, 0.008)	0.92						
Moderate vs. Mild Depression			0.138 (−0.005, 0.282)	0.058						
Severe vs. Mild Depression			0.043 (−0.251, 0.336)	0.78						
Function										
GAF Score					0.004 (−0.001, 0.009)	0.14				
Employment					−0.120 (−0.311, 0.071)	0.22				
Somatic Comorbidities										
Number of Somatic Comorbidities							0.103 (0.059, 0.148)	<0.001 *		
Medications										
First + Second vs. Second-Generation Antipsychotic									−0.272 (−0.768, 0.225)	0.28
>1 Antipsychotic									0.047 (−0.534, 0.629)	0.87
Lithium									−0.370 (−0.626, −0.115)	0.005 *
Anticonvulsant					-				0.257 (0.035, 0.48)	0.023 *
Antidepressant									−0.010 (−0.238, 0.217)	0.93

The symbol * signifies significance *p* < 0.05. CI = confidence interval.

## Data Availability

The data that support the findings of this study are available from the corresponding author upon request and with appropriate data use agreements.

## Supplementary Materials

The following supporting information can be downloaded at: https://www.mdpi.com/article/10.3390/medicina62040761/s1, Table S1: metadata for the contributing studies (N = 1226).
