# Supplementary material for: Demographic and Clinical Correlates of Body Mass Index in Older Age Bipolar Disorder: Results from the GAGE-BD Project
Source: Medicina (Kaunas). 2026 Apr 15;62(4):761. doi: 10.3390/medicina62040761 (PMC13117308; doi:10.3390/medicina62040761)
Supplement: Supplementary file 1 [file medicina-62-00761-s001.zip › medicina-4146110-supplementary.pdf]

Supplemental Table S1
Metadata for the Contributing Studies (N = 1,226)

Full Name of Study	Data Wave	Study Cohort Acronym	Site	Study Design	Total N of Study in Dataset	Location
Atorvastatin for the Treatment of Lithium-Induced Nephrogenic Diabetes Insipidus: A Randomized Controlled Trial	1	Atorvastatin	Lady Davis Institute	RCT	11	Quebec, Canada (NORTH AMERICA)
Treatment Adherence Enhancement in Bipolar Disorder	1	CAE	Case Western Reserve University (multi-site study)	RCT	71	Ohio, USA (NORTH AMERICA)
Dutch Older Bipolar Cohort, Wave 1	1	DOBi1	GGZ inGeest	Observational	39	Amsterdam, Netherlands (EUROPE AND CENTRAL ASIA)
Dutch Older Bipolar Cohort, Wave 2	1	DOBi2	GGZ inGeest	Observational	67	Amsterdam, Netherlands (EUROPE AND CENTRAL ASIA)
Open-label, Prospective Trial of Lamotrigine for Symptoms of Geriatric Bipolar Depression	1	GERI-SAD	Case Western Reserve University	Prospective uncontrolled intervention with Lamotrigine	52	Ohio, USA (NORTH AMERICA)
Geriatric Psychiatry Mood Disorders Research Database	1	GMDD / McLe	McLean Hospital	Observational	10	Massachusetts, USA (NORTH AMERICA)
Health in Men Study	1	HIMS	University of Western Australia	Observational	68	Perth, Australia (EAST ASIA AND PACIFIC)
Dynamic Inflammatory and Mood Predictors of Cognitive Aging in Bipolar Disorder	1	Inflammaging	University of California San Diego	Observational	26	California, USA (NORTH AMERICA)
Asenapine in the Treatment of Older Adults With Bipolar Disorder	1	OPT-BD	Case Western Reserve University	Prospective uncontrolled intervention with Asenapine	15	Ohio, USA (NORTH AMERICA)
Effects of Medication and Medical Morbidity on Brain and Cognition of Old Bipolar Patients	1	TMU 1	Taipei Medical University	Observational	48	Taipei, Taiwan (EAST ASIA AND PACIFIC)

Effects of Medication and Medical Morbidity on Brain and Cognition of Old Bipolar Patients	1	TMU2	Taipei Medical University	Observational	40	Taipei, Taiwan (EAST ASIA AND PACIFIC)
The Effect of Bipolar Disorder and its Comorbidities on Cognition in Older Adults	1	UPMC	University of Pittsburgh Medical Center	Observational	100	Pennsylvania, USA (NORTH AMERICA)
Mood Disorders Research Program Database	1	Yale	Yale School of Medicine	Observational	77	Connecticut, USA (NORTH AMERICA)
Ziprasidone Switching in Response to Adherence in Psychotropic-Related Weight Gain Concerns Among Patients with Bipolar Disorder	1	ZIP-AD	Case Western Reserve University	Prospective uncontrolled intervention with Ziprasidone	9	Ohio, USA (NORTH AMERICA)
**Full Name of Study**	**Data Wave**	**Study Cohort Acronym**	**Site**	**Study Design**	**Total N of Study in Dataset**	**Location**
Several Studies as Described in "Midline Brain Abnormalities Across Psychotic and Mood Disorders"	2	Barcelona(FIDMAG) / Neuroimaging	Hospital Clínic Barcelona / FIDMAG Germanes Hospitalaries	Observational	10	Barcelona, Spain (EUROPE AND CENTRAL ASIA)
Enhancing Adherence and Outcomes in Bipolar Disorder with Abilify Maintena + a Targeted Behavioral Approach to Promote Sustained Adherence and Behavioral Change	2	BD-CAEL	CWRU	Prospective trial with Maintena	14	Ohio, USA (NORTH AMERICA)
Assessment of Clinical Conditions and Biomarkers Related to Psychiatric Outcomes and Cognition in Patients with Bipolar Disorder	2	Belo Horizonte(Bipolar Cordis Est)	UFMG	Observational	33	Belo Horizonte, Brazil (LATIN AMERICA AND THE CARIBBEAN)
The Global Aging and Geriatric Experiments in Bipolar Disorder Database Project	2	FNPHLag	Federal Neuropsychiatric Hospital/ McMaster University	Observational	44	Lagos, Nigeria (SUB-SAHARAN AFRICA)
Acute Pharmacotherapy of Late-Life Mania	2	GERI-BD	CWRU	RCT	218	Ohio, USA (NORTH AMERICA)
Improving Medication Adherence in Hypertensive Individuals with Bipolar Disorder (iTAB-CV) - Phase 2	2	iTAB-CVPilot	CWRU	Prospective cohort design	23	Ohio, USA (NORTH AMERICA)

Mazhar Osman Mood Clinic	2	Konya	Konya - Selçuk University Mazhar Osman Mood Clinic	Observational	12	Konya, Türkiye (EUROPE AND CENTRAL ASIA)
Mood and Metabolism Program Database	2	M&M	Halifax	Clinical database	14	Nova Scotia, Canada (NORTH AMERICA)
Early Diagnosis, Treatment and Prevention of Mood Disorders Targeting the Activated Inflammatory Response System - Bipolar Patients Cohort	2	MOODINFLAME	University of Groningen	Observational	39	Groningen, Netherlands (EUROPE AND CENTRAL ASIA)
Bipolar Disorder Research Program: Cognitive-Behavioral Rehabilitation Versus Treatment as Usual for Bipolar Patients aka "CBT" & C Monohydrate as Adjuvant Therapy for Bipolar Depression aka "Creatine"	2	PROMAN	University of Sao Paulo -PROMAN	RCT	36	São Paulo, Brazil (LATIN AMERICA AND THE CARIBBEAN)
Targeted Training in Illness Management (TTIM) for Individuals with Serious Mental Illness (SMI) and Diabetes Mellitus (DM) Study Protocol	2	TTIM	CWRU	RCT	34	Ohio, USA (NORTH AMERICA)
UTHealth Center of Excellence on Mood Disorders	2	UTH_Houston	Houston	Observational	9	Texas, USA (NORTH AMERICA)
UTHealth Center of Excellence on Mood Disorders	2	UTH_SanAntonio	Houston	Observational	25	Texas, USA (NORTH AMERICA)
UTHealth Center of Excellence on Mood Disorders	2	UTH_UNC	Houston	Observational	1	Texas, USA (NORTH AMERICA)
Structural and Functional Brain Aging in Bipolar Disorder	2	VA_BAI	Veterans Affairs San Diego Healthcare System	Observational	11	California, USA (NORTH AMERICA)
Valencia Outpatient Unit. Cognitive and Social Functioning in Bipolar Disorder	2	VOCS_BD	La Ribera University Hospital. Alzira, Valencia	Observational	70	Valencia, Spain (EUROPE AND CENTRAL ASIA)

Note. RCT = Randomized Controlled Trial
